# Reproducibility of a Short-Form, Multicomponent Dietary Questionnaire to Assess Food Frequency Consumption, Nutrition Knowledge, and Lifestyle (SF-FFQ4PolishChildren) in Polish Children and Adolescents

**DOI:** 10.3390/nu11122929

**Published:** 2019-12-03

**Authors:** Joanna Kowalkowska, Lidia Wadolowska, Jadwiga Hamulka, Natalia Wojtas, Magdalena Czlapka-Matyasik, Witold Kozirok, Monika Bronkowska, Joanna Sadowska, Sylwia Naliwajko, Izabela Dziaduch, Aneta Koronowicz, Ewelina Piasna-Slupecka, Ewa Czeczelewska, Jan Czeczelewski, Malgorzata Kostecka, Anna Dlugosz, Dorota Loboda, Marta Jeruszka-Bielak

**Affiliations:** 1 Department of Human Nutrition, Faculty of Food Sciences, University of Warmia and Mazury in Olsztyn, Sloneczna 45F, 10-718 Olsztyn, Poland; lidia.wadolowska@uwm.edu.pl (L.W.); natalia.ulewicz@uwm.edu.pl (N.W.); 2 Department of Human Nutrition, Institute of Human Nutrition Sciences, Warsaw University of Life Science—SGGW, Nowoursynowska 159C, 02-776 Warsaw, Poland; jadwiga_hamulka@sggw.pl (J.H.); marta_jeruszka_bielak@sggw.pl (M.J.-B.); 3 Institute of Human Nutrition and Dietetics, Poznań University of Life Sciences, 31 Wojska Polskiego St., 60-624 Poznań, Poland; magdalena.matyasik@up.poznan.pl; 4 Department of Commodity and Quality Management, Faculty of Entrepreneurship and Quality Science, Gdynia Maritime University, 81-87 Morska Street, 81-225 Gdynia, Poland; w.kozirok@wpit.umg.edu.pl; 5 Department of Human Nutrition, Faculty of Biotechnology and Food Science, Wrocław University of Environmental and Life Sciences, Chełmońskiego 37, 51-630 Wroclaw, Poland; monika.bronkowska@upwr.edu.pl; 6 Department of Human Nutrition Physiology, Faculty of Food Sciences and Fisheries, West Pomeranian University of Technology in Szczecin, Papieża Pawła VI 3, 71-459 Szczecin, Poland; joanna.sadowska@zut.edu.pl (J.S.); izabela.dziaduch@zut.edu.pl (I.D.); 7 Department of Bromatology, Faculty of Pharmacy with the Division of Laboratory Medicine, Medical University of Bialystok, Mickiewicza 2D, 15-222 Bialystok, Poland; sylwia.naliwajko@umb.edu.pl; 8 Department of Human Nutrition, Faculty of Food Technology, University of Agriculture in Krakow, Balicka 122, 30-149 Krakow, Poland; aneta.koronowicz@gmail.com (A.K.); piasna.ewelina@gmail.com (E.P.-S.); 9 Department of Nursing, Faculty of Health Sciences, Collegium Mazovia Innovative Higher School in Siedlce, Sokolowska 161, 08-110 Siedlce, Poland; eczeczelewska@mazovia.edu.pl; 10 Human Nutrition Laboratory, Faculty of Physical Education and Sport in Biala Podlaska, Jozef Pilsudski University of Physical Education, Warsaw, Akademicka 2, 21-500 Biala Podlaska, Poland; jan.czeczelewski@awf-bp.edu.pl; 11 Department of Chemistry, Faculty of Food Science and Biotechnology, University of Life Sciences, 15 Akademicka Street, 20-950 Lublin, Poland; kostecka.malgorzatam@gmail.com; 12 Faculty of Chemical Technology and Engineering, University of Technology and Life Sciences in Bydgoszcz, Seminaryjna 3, 85-326 Bydgoszcz, Poland; anna.dlugosz@utp.edu.pl; 13 Institute of Health, University of Economy in Bydgoszcz, Garbary 2, 85-229 Bydgoszcz, Poland; dorota.loboda@byd.pl

**Keywords:** food frequency questionnaire, reproducibility, reliability, eating behaviors, meal consumption, nutrition knowledge, physical activity, schoolchildren

## Abstract

The aim of the study was to assess the reproducibility of a short-form, multicomponent dietary questionnaire (SF-FFQ4PolishChildren) in Polish children and adolescents. The study involved 437 children (6–10 years old) and 630 adolescents (11–15 years old) from rural and urban areas of Poland. The self-administered questionnaire was related to nutrition knowledge, dietary habits, active/sedentary lifestyle, self-reported weight and height, and socioeconomic data. The questionnaire was completed with a two-week interval—twice by parents for their children (test and retest for children), twice by adolescents themselves (adolescent’s test and retest) and once by adolescents’ parents (parent’s test). The strength of agreement measured using the kappa statistic was interpreted as follows: 0–0.20 slight, 0.21–0.40 fair, 0.41–0.60 moderate, 0.61–0.80 good, and 0.81–1.00 excellent. Regarding the frequency of consumption of food items and meals, kappa statistics were 0.46–0.81 (the lowest: fruit/mixed fruit and vegetable juices; the highest: Energy drinks) in test–retest for children, 0.30–0.54 (fruit/mixed fruit and vegetable juices; breakfast, respectively) in adolescent’s test–retest, 0.27–0.56 (the lowest: Sweets, fruit, dairy products; the highest: Breakfast) in adolescent’s test and parent’s test. Lower kappa statistics were found for more frequently consumed foods (juices, fruit, vegetables), higher kappa statistics were found for rarely consumed foods (energy drinks, fast food). Across study groups, kappa statistics for diet quality scores were 0.31–0.55 (pro-healthy diet index, pHDI) and 0.26–0.45 (non-healthy diet index, nHDI), for active/sedentary lifestyle items they were 0.31–0.72, for components of the Family Affluence Scale (FAS) they were 0.55–0.93, for BMI categories (based on self-reported weight and height) they were 0.64–0.67, for the nutrition knowledge (NK) of adolescents the kappa was 0.36, for the nutrition knowledge of children’s parents it was 0.62. The Spearman’s correlations for diet quality scores were 0.52–0.76 (pHDI) and 0.53–0.83 (nHDI), for screen time score they were 0.45–0.78, for physical activity score they were 0.51–0.77, for the FAS score they were 0.90–0.93, and for the NK score they were 0.68–0.80. The questionnaire can be recommended to evaluate dietary and lifestyle behaviors among children and adolescents.

## 1. Introduction

Dietary and lifestyle behavior evaluation in children and adolescents is crucial to develop and implement effective policies preventing dietary-related diseases and ensuring public health in the future. Lifestyle habits affect health and are shaped at an early age and track throughout adolescence into adulthood [1,2,3].

Dietary assessment in children and adolescents can be more difficult than in adults [4]. In children, low cognitive abilities, e.g., limited knowledge of food, memory, conceptualization of frequency, may lead to substantial misreporting and, therefore, dietary reporting is conducted by proxies, typically parents or caregivers [4,5,6]. Adolescents are characterized by a high day-to-day variability in diet [5]. They may also perceive long questionnaires as boring or tiring and be less interested in giving valid answers [6]. Short tools allowing quick, easy, low-cost, and reliable assessment of dietary and lifestyle behaviors can be more useful in large epidemiological research and more suitable for children and adolescents than long questionnaires [4,7,8,9]. Shorter questionnaires are described as potentially more advantageous if they can accurately discriminate people with low and high food consumption and identifying groups at risk [10].

Each newly developed or adapted tool should be tested for reproducibility and validity in the target population [10,11,12]. A range of measurement errors affected the reproducibility (random errors) and validity (systematic errors) of dietary methods. Since it is impossible to eliminate them completely, it is important to identify possible sources and to assess the level of errors [12]. For example, one of the common errors in dietary assessment methods is misreporting of foods and/or portion sizes consumed over a specified period of time, leading to underestimation or overestimation of energy and nutrient intake [4,7,12,13].

To the best of our knowledge, there are only a few food frequency questionnaires (FFQs) in Poland for which reproducibility or relative validity among children or adolescents is assessed [14,15,16]. The relative validity of a semi-quantitative FFQ against repeated 24-h dietary recalls was evaluated in Polish children aged 3 years old, showing overestimation of energy and nutrient intake by the FFQ (e.g., median of differences in energy intake between the two methods was 255.4 kcal, and in the range of 7.0–31.0 g for intake of macronutrients) [14]. A non-quantitative FFQ (KomPAN^®^) was tested in Polish adolescents and adults aged 15–65 years, showing moderate to very good reproducibility of the questionnaire (e.g., kappa statistic for food items was in the range of 0.62–0.84 for the interviewer-administered questionnaire and 0.5–0.78 for the self-administered questionnaire in healthy subjects) [15]. Another non-quantitative FFQ (62-item FFQ-6) was tested in Polish females aged 13–21 years, demonstrating good or very good reproducibility for most food items and acceptable-to-good reproducibility of identification of dietary patterns (e.g., the Spearman correlations were more than 0.50 for 57 out of 62 food items, and for dietary pattern scores were in the range of 0.48–0.84 in the total sample) [16]. There is a lack of tested questionnaires developed for children and younger adolescents. Therefore, the aim of this study was to assess the reproducibility of a short-form, multicomponent dietary questionnaire to assess food frequency consumption, nutrition knowledge, and lifestyle (SF-FFQ4PolishChildren) in Polish schoolchildren aged 6–15 years.

## 2. Materials and Methods

### 2.1. Ethical Approval

The study was approved by the Bioethics Committee of the Faculty of Medical Sciences, University of Warmia and Mazury in Olsztyn in 17 June 2010 (resolution no. 20/2010). Informed consent was obtained from parents or legal guardians of schoolchildren.

### 2.2. Study Design

**Figure 1:**
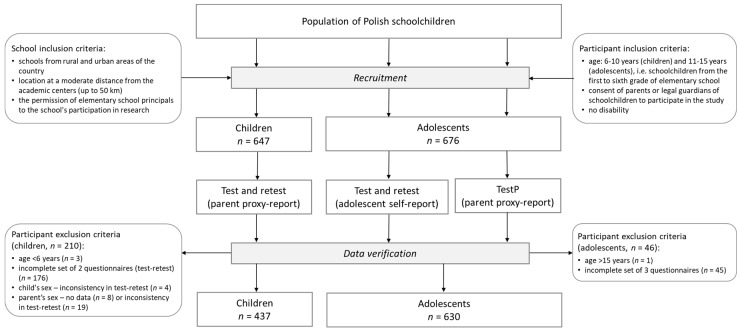
Study design and data collection.

The study was conducted in Poland in autumn 2016. The recruitment was carried out in elementary schools (not randomly selected) located across the whole country, covering rural and urban areas (Figure S1). Inclusion criteria for schools were location at a moderate distance from the academic centers (up to 50 km) and the permission of school principals for the school’s participation in research (Figure 1). Schoolchildren from the first to sixth grade of elementary schools were invited to take part in the study. The self-administered questionnaire was administered in a paper format. The questionnaire was completed with a two-week interval—twice by parents for their children (test–retest for children), twice by adolescents themselves (adolescent’s test–retest), and once by the adolescents’ parents (testP) (Figure 1). In line with previous studies [17,18,19,20,21], the two-week interval was chosen as long enough to avoid recalling previous responses, but short enough to avoid real changes in dietary behaviors, lifestyle, or nutrition knowledge in schoolchildren. Dietary behaviors of schoolchildren, especially adolescents, can change more rapidly than adults [4,5,6].

### 2.3. Participants

Initially, 1323 subjects were recruited as a convenience sample, and 256 respondents (19.3% of the sample) were excluded due to: respondent’s age—under 6 years (*n* = 3) or over 15 years (*n* = 1), incomplete sets of questionnaires (i.e., less than two for children or less than three for adolescents) (*n* = 221), child’s sex—inconsistency in test–retest (*n* = 4), parent’s sex—no data (*n* = 8) or inconsistency in test–retest (*n* = 19) (Figure 1). Finally, in total 1067 respondents were included in the study—437 children (6–10 years old) and 630 adolescents (11–15 years old).

### 2.4. A Short-Form, Multicomponent Dietary Questionnaire (SF-FFQ4PolishChildren)

The SF-FFQ4PolishChildren is a self-administered tool developed for schoolchildren by Kowalkowska, Wadolowska, and Hamulka in 2015 and previously published by Hamulka et al. [22]. The questionnaire consisted of a total of 44 items regarding: Dietary habits (11 items), nutrition knowledge (18 items), active/sedentary lifestyle (3 items), demographics (4 items), the Family Affluence Scale (FAS) components (6 items), anthropometric data (2 items). The reference period of the questionnaire was the previous 12 months.

### 2.5. Dietary Habits

The habitual frequency of eating two meals (breakfast, a meal at school) and the consumption of nine food items (dairy products, fish, fast food, sweetened soft drinks, fruit/mixed fruit and vegetable juices, energy drinks, vegetables, fruit, sweets) were collected. Respondents reported their breakfast consumption by choosing one of four categories: Less than once a week, 1–3 times/week, 4–6 times/week, every day. A meal at school was considered as the second eating episode of the school day (e.g., lunch, second breakfast). Respondents reported its consumption choosing one of four categories: Less than once a week, 1–2 times/week, 3–4 times/week, every school day (5 times/week). For food items, respondents could choose one of seven consumption frequency categories (converted into daily frequency, times/day): Never/almost never (0), less than once a week (0.06), once a week (0.14), 2–4 times/week (0.43), 5–6 times/week (0.79), every day (1), a few times a day (2) [22].

To evaluate overall diet quality, a pro-healthy diet index (pHDI) and a non-healthy diet index (nHDI) were established based on previous knowledge and other studies [15,23]. The diet quality scores were created a priori by summing the consumption frequencies (times/day) of the following food items: The pHDI—dairy products, fish, vegetables, fruit; the nHDI—fast food, sweetened soft drinks, energy drinks and sweets [22]. Each diet quality score was expressed in % points and categorized as follows: Low (0–33.32% points), moderate (33.33–66.65% points), high (66.66–100% points).

### 2.6. Nutrition Knowledge

Nutrition knowledge (NK) was assessed based on 18 questions with five response categories, including “I don’t know” [22]. The questions were developed based on a questionnaire described by Whati et al. [24] and adapted to Polish conditions and education [25]. The NK score was calculated for each respondent by summing the points obtained from correct answers (each for 1 point). Respondents were classified into three NK levels: Low (0–5 points), moderate (6–12 points), high (13–18 points). In the children’s group, NK was only assessed for their parents.

### 2.7. Active/Sedentary Lifestyle

Three questions related to screen time (ST) and physical activity (PA) at school and at leisure time were applied, which had been previously developed for 15–65-year-olds (the KomPAN^®^ questionnaire) [15,23] and adopted for younger respondents. Respondents reported ST, choosing one of six categories (hours/day): <2, 2 to <4, 4 to <6, 6 to <8, 8 to <10, and ≥10. Scores from 0 points for the shortest time (<2 h/day) to 5 points for the longest ST (≥10 h/day) were assigned [22]. For PA at school and PA at leisure time, respondents could choose one of three categories for each type of PA: Low, moderate, high. Based on both types of PA, the total PA level was evaluated by assigning scores from 0 to 5 points (Table 1). Respondents were classified into three categories of the PA level: Low (0–1 points), moderate (2–4 points), and high (5 points).

### 2.8. The Family Affluence Scale Components

The socioeconomic assessment was based on six questions of FAS (version III) described by the Polish team of the Health Behavior of School-Aged Children (HBSC) international study [26] (Table 2). The FAS score was calculated by summing points assigned for the categories selected by a respondent in each of the questions and ranged from 0 to 9 points [26].

### 2.9. Anthropometric Data

Data on self-reported height and body weight were collected, and the body mass index (BMI) was calculated. According to age-sex-specific international standards for children and adolescents [27], respondents were classified into three BMI-for-age categories: Thinness, normal weight, and overweight.

### 2.10. Statistical Analysis

Means with 95% confidence interval (CI) and percentage distribution of participant characteristics were calculated. Normality of the distribution of continuous variables in the total sample, boys and girls, as well as rural and urban residents was checked by the Kolmogorov–Smirnov test. Urban residents were respondents who indicated one of the following categories of place of residence: “town” or “city (≥100,000 inhabitants)”. The test–retest reproducibility of the questionnaire was assessed as follows: (i) cross-classification analysis and the kappa statistic were calculated for 25 variables: NK level, dietary habits (13 variables), lifestyle (4 variables), FAS components (six variables), BMI categories; (ii) the Spearman’s rank correlation coefficient was calculated for six variables (all in points) in the total sample as well as by sex and place of residence: NK score, pHDI, nHDI, ST score, PA score, FAS; (iii) means were calculated for the same six variables (all in points) in the total sample and by sex and place of residence and then compared between test and retest using Wilcoxon signed-rank test (for two dependent samples); (iv) the Bland–Altman method was used for diet quality scores (pHDI, nHDI) to assess an agreement between test and retest (or parent’s test) in the total sample and by sex groups [28]. Mean difference between test and retest, 95% limits of agreement (LOA), and the Bland–Altman index (percentage of respondents beyond LOA) were calculated. The Bland–Altman index ≤5% indicated good test–retest reproducibility of the diet quality score [28,29]. The strength of correlation was interpreted as follows: 0–0.29 fair, 0.30–0.49 moderate, 0.50–0.69 good, and 0.70–1.00 very good. The strength of agreement measured using the kappa statistic was interpreted as follows: 0–0.20 slight, 0.21–0.40 fair, 0.41–0.60 moderate, 0.61–0.80 good, and 0.81–1.00 excellent [30].

All statistical analyses were performed using STATISTICA software (version 12.0 PL; StatSoft Inc., Tulsa, OK, USA; StatSoft, Cracow, Poland), and *p* ≤ 0.05 was considered significant.

## 3. Results

### 3.1. Participant Characteristics

Participant characteristics in the first administration of the questionnaire are shown in Table 3.

### 3.2. Nutrition Knowledge

Cross-classification agreement of the NK level was 85.0% for children’s parents and 72.7% for adolescents (Table 4). The kappa values were 0.62 and 0.36, respectively (Table 5). The Spearman’s correlations for NK score were 0.80 and 0.68, respectively (all <0.05) (Table 6). Correlations for NK score and five other variables (i.e., pHDI, nHDI, ST score, PA score, FAS) by sex and place of residence are shown in Table S1, while comparison of mean values of six variables between test and retest (or parent’s test) in the total sample and by sex and place of residence are shown in Table S2.

### 3.3. Dietary Habits

The proportion of respondents classified into the same frequency category was 59.0–98.4% (the lowest: Fruit/mixed fruit and vegetable juices; the highest: Energy drinks) for children, 42.9–80.1% (fruit/mixed fruit and vegetable juices; energy drinks, respectively) in the adolescent’s test–retest, 41.1–83.9% (sweetened soft drinks; energy drinks, respectively) in the adolescent’s test and parent’s test (Table 4). Kappa was 0.46–0.81 (fruit/mixed fruit and vegetable juices; energy drinks, respectively) in test–retest for children, 0.30–0.54 (fruit/mixed fruit and vegetable juices; breakfast, respectively) in the adolescent’s test–retest, 0.27–0.56 (the lowest: Sweets, fruit, dairy products; the highest: Breakfast) in the adolescent’s test and parent’s test (Table 5).

**Figure 2:**
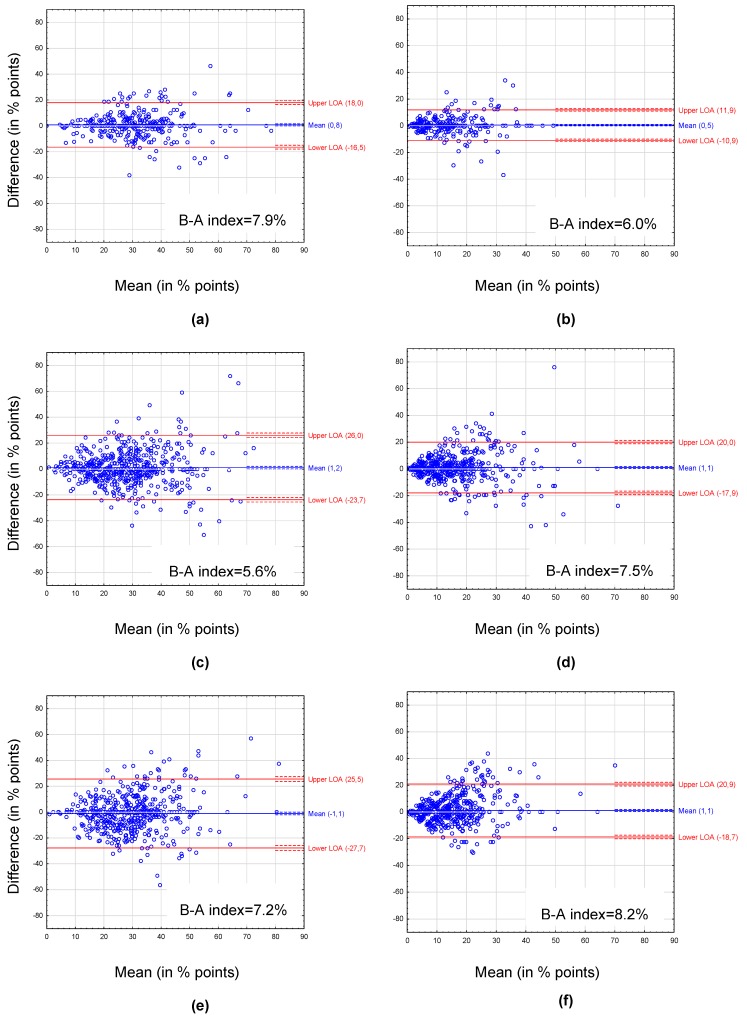
Bland–Altman plots for the pro-healthy diet index (pHDI; left panel) and the non-healthy diet index (nHDI; right panel) between the first and the second administration of the questionnaire: (**a**) pHDI in children aged 6–10 years (test and retest), (**b**) nHDI in children aged 6–10 years (test and retest), (**c**) pHDI in adolescents aged 11–15 years (test and retest), (**d**) nHDI in adolescents aged 11–15 years (test and retest), (**e**) pHDI in adolescents aged 11–15 years and their parents (test and testP), (**f**) nHDI in adolescents aged 11–15 years and their parents (test and testP). Mean—mean difference between the first and the second administration of the questionnaire (blue solid line) with 95% CI (dashed lines). LOA—95% limits of agreement between the first and the second administration of the questionnaire (red solid lines) with 95% CI (dashed lines). B-A index—the Bland–Altman index calculated as percentage of respondents beyond LOA.

Cross-classification agreement for pHDI was 79.5% for children, 75.8% in adolescent’s test–retest, and 69.0% in the adolescent’s test and parent’s test; for nHDI: 96.8%, 92.0%, and 92.3%, respectively (Table 4). Kappa values were 0.55, 0.44, 0.31 for pHDI and 0.45, 0.35, 0.26 for nHDI, respectively (Table 5). The Spearman’s correlations were 0.76, 0.63, 0.52 for pHDI and 0.83, 0.68, 0.53 for nHDI, respectively (all <0.05) (Table 6). The Bland–Altman plots showed mean difference ranged from −1.1% points for pHDI in the adolescent’s test and parent’s test to 1.2% points for pHDI in the adolescent’s test–retest (Figure 2). The Bland–Altman index ranged from 5.6% for pHDI in the adolescent’s test–retest to 8.2% for nHDI in the adolescent’s test and parent’s test. Results of the Bland–Altman methods by sex groups are shown in supplementary materials (Figures S2 and S3).

### 3.4. Active/Sedentary Lifestyle

For PA and ST, cross-classification agreement was 83.8–86.9% for children, 64.8–74.8% in the adolescent’s test–retest, 59.9–65.3% in the adolescent’s test and parent’s test (Table 4). Kappa values were 0.68–0.72 for children, 0.46–0.54 in the adolescent’s test–retest, 0.31–0.40 in the adolescent’s test and parent’s test (Table 5). The Spearman’s correlations for ST score were 0.78 for children, 0.58 in the adolescent’s test–retest, 0.45 in the adolescent’s test and parent’s test, while the correlations for PA score were 0.77, 0.71 and 0.51, respectively (all <0.05) (Table 6).

### 3.5. Socioeconomic Data

Regarding FAS components, cross-classification agreement was 87.1–97.5% for children, 83.7–95.8% in the adolescent’s test–retest and 75.6–95.5% in the adolescent’s test and parent’s test (Table 4). The kappa statistics were 0.79–0.93 for children, 0.69–0.89 in the adolescent’s test–retest, 0.55–0.87 in the adolescent’s test and parent’s test (Table 5). The Spearman’s correlations for the FAS score were 0.93, 0.91 and 0.90, respectively (all <0.05) (Table 6).

### 3.6. Anthropometric Data

Cross-classification agreement of the BMI categories was 89.4% for children, 90.2% in the adolescent’s test–retest, 92.1% in the adolescent’s test and parent’s test (Table 4). The kappa values were 0.67, 0.64, and 0.65, respectively (Table 5).

## 4. Discussion

The study showed moderate-to-excellent reproducibility, measured with kappa statistic, for all 25 analyzed items and scores in children (with parents as proxy reporters), 17 of 25 of items/scores (68%) in adolescents and 10 of 24 of items/scores (42%) in the adolescent’s test and parent’s test. In children, the questionnaire demonstrated moderate reproducibility for most foods and diet quality scores, while good-to-excellent for other items/scores related to meal consumption frequency, nutrition knowledge, active/sedentary lifestyle, BMI categories, and FAS components. For adolescents, the reproducibility was fair-to-moderate for all dietary habits, nutrition knowledge, active/sedentary lifestyle, and good-to-excellent for other items. When the adolescent’s test was compared to the parent’s test, the reproducibility was slightly weaker than when the adolescent’s test and retest were compared. This may be explained by the growing independence of adolescents and lower parental knowledge regarding the pupils’ diets due to spending less time together [6].

The test–retest reproducibility for food items found in the present study in children (agreement: 59.0–98.4%, kappa: 0.46–0.81) and adolescents (42.9–80.1%, kappa: 0.30–0.44) was similar or higher than previously reported [31,32,33,34,35,36,37,38]. Parent-administered semi-quantitative FFQs to assess children’s diet demonstrated cross-classification agreement, which ranged from 33–69% in Danish children [32] and 62.1–99.4% in Spanish children [37], but it ranged from 44–82% in New Zealand children using a short non-quantitative FFQ [35]. Lower reproducibility of food consumption frequency was found in children from six European countries (kappa: 0.23–0.68) [39]. For adolescents, the cross-classification agreement found in other reproducibility studies was similar or lower than our findings and ranged from: 29–58% in Italian adolescents [33], 36–55% in Norwegian adolescents [34], 37–87% in Belgian adolescents [36], 45–77% in Danish adolescents [31] and 46–88% in New Zealand adolescents [38], while the kappa values for foods obtained in adolescents were higher compared to our findings: 0.21–0.66 [33], 0.23–0.71 [31], 0.43–0.70 [36].

The present study showed lower reproducibility for more frequently consumed foods (juices, fruit, vegetables) and higher reproducibility for rarely consumed foods (energy drinks, fast food). Similarly, lower test–retest agreement was demonstrated for frequently consumed foods (e.g., white bread, wholemeal bread, fruit juices) and higher agreement for rarely consumed foods (e.g., porridge, fish, energy drinks) in Danish adolescents [31] and Polish adolescents and adults [15]. Estimating the consumption frequency of foods eaten less frequently or never could be easier than those eaten more often and included in various dishes and meals [11,31].

Since the present study showed relatively low reproducibility for certain food items, e.g., fruit and vegetables, the use of diet quality scores (developed based on the questionnaire) seems to be more appropriate than the use of single food items. The advantage of using diet scores, as a comprehensive approach, and their utility to study diet–health relationships were highlighted previously [40,41]. In the present study, the Bland–Altman plots showed very good test–retest reproducibility of diet quality scores at the group level (mean differences from −1.1% to 1.2% points), but moderate reproducibility at the individual level—the 95% LOAs were relatively wide, and values of the Bland–Altman index were slightly higher than 5% [28,29]. Better agreement between test and retest of the questionnaire was observed at low values of diet quality scores than at moderate or high values of pHDI or nHDI. These findings may indicate better test–retest reproducibility of the questionnaire in terms of dietary behaviors in children and adolescents with stable but restrictive eating habits (consuming key foods, healthy or unhealthy, with low frequency and/or consuming only a few selected foods with higher frequency). Better test–retest reproducibility of FFQs for foods consumed rarely or never has been reported in other studies [15,16,31]. The differences between both administrations of the questionnaire were more scattered in adolescents than in children, and the most between the adolescent’s test and parent’s test, which confirms the results of other statistical analyses. Furthermore, most of the mean differences showed an overestimation of diet quality scores in the test compared to the retest. Negative mean differences were found only for the pHDI between the adolescent’s test and the parent’s test in the total sample as well as in sex groups, which may indicate an overestimation of pHDI in parental reporting compared to adolescent self-reporting. A literature review of McPherson et al. [42] showed that the results from the first administration of the questionnaire usually tend to be higher compared to subsequent administrations. In turn, adolescents are characterized by less structured food habits that can change rapidly, eating more out-of-home and growing independence from parents, which can lead to less knowledge of parents about their children’s diet [4,6]. A parental misperception of the child’s diet quality—an overestimation of “healthy” food choices and/or underestimation of less “healthy” food consumption—has been noted in other research [11,43]. All this together can explain the greater discrepancies between test and retest in adolescents, as well as between adolescents and their parents, than in both administrations of the questionnaire in children. Compared to our findings obtained by various statistics (pHDI: Agreement: 69.0–79.5%, kappa: 0.31–0.55, *r* = 0.52–0.76; nHDI: Agreement: 92.0–96.8%, kappa: 0.26–0.45, *r* = 0.53–0.83), other studies demonstrated similar test–retest reproducibility for FFQ-based diet scores in: Flemish children (kappa: 0.61; *r* = 0.88) [44], New Zealand adolescents (agreement: 60%) [17], Norwegian adolescents (agreement: 87.6%; kappa: 0.465) [40] and Norwegian parents of toddlers (kappa: 0.52; *r* = 0.80) [18].

For NK levels, good reproducibility in children’s parents and fair reproducibility in adolescents was found. For NK score, the strength of the Spearman’s correlation between test and retest was very good in children’s parents and good in adolescents. Means of NK score were different in adolescent’s test–retest only. It may be speculated that the first administration of the questionnaire created interest in this knowledge, causing an increase in the adolescents’ NK in the second administration. Relatively weak reproducibility for single NK items was found in Australian schoolchildren (intra-class correlation (ICC): 0.16–0.36) [45], U.S. adolescents (kappa: 0.30–0.56) [19]. High reproducibility of NK was demonstrated in Belgian schoolchildren (NK score: ICC = 0.76) [20], Italian children and adolescents (Pearson’s correlation coefficient (*r*): 0.87) [18] or adolescents (*r* = 0.80) [46].

Active/sedentary lifestyle items showed high reproducibility, especially in children. In adolescents, the agreement was moderate, but fair when the adolescent’s test was compared to parental reporting. For ST score, the strength of the Spearman’s correlation between test and retest was very good in children and good in adolescents, but moderate when the adolescent’s test was compared to parental reporting. For PA score, the strength of correlation was very good in both children and adolescents and good between the adolescent’s test and parent’s test. Similar results of correlations between both administrations of the questionnaire were observed in sex groups as well as in rural and urban residents. High test–retest reproducibility was demonstrated for PA in U.S. adolescents (agreement: 66–89%) [19], PA and lifestyle in Italian children and adolescents (*r* = 0.70) [21], or adolescents (*r* = 0.88) [46]. Parental awareness of children’s PA was low; most parents overestimated their child’s PA [47]. Regarding sedentary behaviors, among Norwegian schoolchildren test–retest reproducibility was moderate for weekly scores (*r* = 0.66–0.73): TV/DVD use, computer/electronic game use, and total ST [48] and was slightly lower for single ST items (*r* = 0.50–0.65) [49].

For FAS components and the FAS score, good-to-excellent reproducibility was found across study groups, except for the component regarding family computers in the adolescent’s test and parent’s test (kappa: 0.55). Similar results were found in a previous validation study in Polish adolescents and their parents [26]. For FAS components, moderate-to-excellent agreement (kappa: 0.58–0.83) between the adolescents and parents and excellent (0.83–0.95) in the adolescent’s test–retest were found with the lowest reproducibility for family computers [26]. In 11-year-olds and their parents from six European countries, validation of FAS II (with the same three components as in FAS III) showed high reproducibility of FAS components with relatively low agreement for family computers (kappa for six countries: 0.68, Poland: 0.48) [50].

For BMI categories calculated from self-reported data, high cross-classification agreement (89.4–92.1%) and good inter-rater reliability (kappa: 0.64–0.67) were shown. The reproducibility was very similar across study age groups and between the adolescent’s test and parent’s test. Given the overall importance of body image concern, dieting behaviors, peer and media influence in adolescence [6], greater differences between the values reported by adolescents and their parents were expected. Parental reports of weight and height to assess children’s BMI are cost-efficient and often used in large-scale surveys [51,52]. However, comparing our findings with the results of other research is difficult because most of the previous studies referred to self-reported values compared to measured values [51,52,53].

### Strengths and Limitations

Several strengths and limitations of this study should be emphasized for future research. The study was conducted using a large sample of over a thousand 6–15-year-old subjects, greater than samples described in other reproducibility studies among schoolchildren [31,33,38,46]. Although this was not a representative national sample, it covered rural and urban areas in all macro-regions of the country [54]. To describe adolescents, the study involved parents and adolescents themselves, which allowed the reproducibility of the data collected in the retest among those groups to be verified and a better approach for adolescents to be selected. Since another strength of the study is assessing the reproducibility of single questions as well as total scores, our findings show more options of the questionnaire application. Moreover, a variety of statistical methods were used to strengthen the conclusions and facilitate comparison of our results with others.

As the study limitation, involving only parents to describe children’s dietary and lifestyle behaviors should be considered. However, this technique of data collection is in line with the recommendations for dietary assessment using questionnaires in children [5,55,56]. Furthermore, only self-reported data were collected with this questionnaire, so misreporting and social desirability biases should be taken into account [12]. Misreporting is one of the most commonly reported measurement errors in dietary assessment methods [4,7,12,13]. Respondents may misreport certain foods systematically—those with low consumption of “healthy” foods may tend to overreport their intake, while those with high consumption of “unhealthy” foods may tend to underreport them [12]. Similarly, possibility of incorrect perception of body image in some respondents and a social desirability bias may not contribute to a lower reproducibility of the classification to BMI category. Such respondents may under-report their body weight and over-report their height to the same extent in both administrations of the questionnaire. Although testing reproducibility of a questionnaire reflects random errors, validation of the tool provides information on systematic errors that are more difficult to control [12]. Therefore, further study to validate the questionnaire against biomarkers and/or other dietary methods as the reference as well as using measured anthropometric data should be conducted.

## 5. Conclusions

The study showed moderate-to-excellent reproducibility of the questionnaire items and scores with some exceptions. Worse reproducibility was found for more frequently consumed foods, such as juices, fruit, and vegetables. To describe adolescents, the reproducibility was better when the questionnaire was completed by adolescents than parents. The questionnaire can be recommended to evaluate dietary and lifestyle behaviors among children and adolescents.

## Figures and Tables

**Table 1:** Categorizing and scoring of the total physical activity level [22].

Physical Activity at School	Physical Activity at Leisure Time		
	Low	Moderate	High
Low	Low (0 points)	Low (1 points)	Moderate (2 points)
Moderate	Low (1 points)	Moderate (3 points)	Moderate (4 points)
High	Moderate (2 points)	Moderate (4 points)	High (5 points)

**Table 2:** Components of the Family Affluence Scale [26].

	Question	Response Categories
1.	How many computers, laptops, or tablets does your family own?	none (0 points); one (1 point); two (2 points); more than two (2 points)
2.	Does your family own a car, van, or truck?	no (0 points); yes, one (1 point); yes, two or more (2 points)
3.	Does your family have a dishwasher?	no (0 points); yes (1 point)
4.	Do you have your own bedroom?	no (0 points); yes (1 point)
5.	How many bathrooms (room with a bath or shower) are in your home?	none (0 points); one (1 point); two (2 points); more than two (2 points)
6.	Does your home have an outdoor space attached (e.g., garden)?	no (0 points); yes (1 point)

**Table 3:** Characteristics of children and adolescents in the first administration of the SF-FFQ4PolishChildren questionnaire.

Variables	Children Aged 6–10 Years (Questionnaire Filled Out by A Parent)		Adolescents Aged 11–15 Years (Questionnaire Filled Out by An Adolescent)	
	*n*	%	*n*	%
Sample size	437		630	
Sex				
boys	211	48.3	325	51.6
girls	226	51.7	305	48.4
Age (years) ^1^	437	8.0 (7.9; 8.1)	630	12.5 (12.4; 12.6)
Residence	437		630	
rural	231	52.9	293	46.5
urban ^2^	206	47.1	337	53.5
FAS (points) ^1^	431	6.2 (6.0; 6.4)	622	6.8 (6.7; 6.9)
Nutrition knowledge score (points) ^1,3^	436	10.4 (10.1; 10.6)	626	7.2 (6.9; 7.4)
Nutrition knowledge level ^3^	436		626	
low	28	6.4	191	30.5
moderate	310	71.1	417	66.6
high	98	22.5	18	2.9
pHDI (%points) ^1^	433	31.5 (30.3; 32.8)	628	29.0 (27.9; 30.1)
pHDI category	433		628	
low	252	58.2	417	66.4
moderate	175	40.4	204	32.5
high	6	1.4	7	1.1
nHDI (%points) ^1^	436	13.0 (12.2; 13.9)	627	15.1 (14.2; 16.0)
nHDI category	436		627	
low	416	95.4	577	92.0
moderate	20	4.6	49	7.8
high	0	0.0	1	0.2
Screen time score (points) ^1^	437	0.4 (0.4; 0.5)	629	0.9 (0.8; 1.0)
Screen time category	437		629	
<2 h/day	278	63.6	262	41.7
2 to <4 h/day	134	30.7	238	37.8
4 to <6 h/day	22	5.0	90	14.3
6 to <8 h/day	0	0.0	19	3.0
8 to <10 h/day	2	0.5	10	1.6
≥10 h/day	1	0.2	10	1.6
Physical activity score (points) ^1^	437	3.2 (3.0; 3.3)	629	3.4 (3.3; 3.5)
Physical activity level	437		629	
low	73	16.7	99	15.7
moderate	304	69.6	378	60.1
high	60	13.7	152	24.2
Physical activity at school	437		629	
low	74	16.9	68	10.8
moderate	284	65.0	316	50.2
high	79	18.1	245	39.0
Physical activity at leisure time	437		629	
low	31	7.1	81	12.9
moderate	219	50.1	284	45.2
high	187	42.8	264	42.0
BMI-for-age	415		595	
thinness	76	18.3	81	13.6
normal weight	263	63.4	425	71.4
overweight	76	18.3	89	15.0

^1^ mean and 95% confidence interval (CI); ^2^ urban residents—respondents who indicated one of the following categories of place of residence: “town” or “city (≥100,000 inhabitants)”; FAS—the Family Affluence Scale composed of six questions and ranged from 0–9 points [26]; nutrition knowledge score—evaluated based on 18 questions and ranged 0–18 points [22]; nutrition knowledge level—assessed in three categories: Low (0–5 points), moderate (6–12 points), high (13–18 points); ^3^ in a group of 6–10-year-old children, nutrition knowledge was assessed in their parents; pHDI (% points)—a pro-healthy diet index composed of four questions (dairy products, fish, vegetables, fruit) and ranged from 0–100 points [22]; pHDI category—low (0–33.32% points), moderate (33.33–66.65% points), high (66.66–100% points); nHDI (% points)—a non-healthy diet index composed of four questions (fast food, sweetened soft drinks, energy drinks, sweets) and ranged from 0–100 points [22]; nHDI category—low (0–33.32% points), moderate (33.33–66.65% points), high (66.66–100% points); screen time score—based on a single question with six response categories and ranged from 0–5 points [22]; physical activity score—based on two questions: Physical activity at school and physical activity at leisure time, and ranged from 0–5 points [22]; physical activity level—assessed in three categories: Low (0–1 points), moderate (2–4 points), high (5 points); BMI-for-age—the age-sex-specific body mass index calculated using self-reported height and weight and assessed in three categories [27].

**Table 4:** Agreement of classification in test and retest of the SF-FFQ4PolishChildren questionnaire (%).

Variables	Cat. ^1^	Children Aged 6–10 Years				Adolescents Aged 11–15 years							
		Parent (Test–Retest)				Adolescent (Test–Retest)				Adolescent (Test) & Parent (TestP)			
		*n*	Total Agreement	Misclassification		*n*	Total Agreement	Misclassification		*n*	Total Agreement	Misclassification	
				±1 Cat.	±2 Cat. or More			±1 Cat.	±2 Cat. or More			±1 Cat.	±2 Cat. or More
Nutrition knowledge level ^2^	3	432	85.0	14.8	0.2	615	72.7	27.3	0.0	628	NA		
Dietary habits													
Breakfast	4	436	88.8	7.8	3.4	629	77.3	17.8	4.9	628	80.3	12.6	7.2
Meal at school	4	435	95.4	3.9	0.7	629	79.8	16.2	4.0	626	81.6	14.7	3.7
Dairy products	7	435	68.0	24.8	7.1	630	51.3	33.8	14.9	627	44.3	34.4	21.2
Fish	7	436	77.1	19.3	3.7	628	57.3	31.5	11.1	627	50.9	37.0	12.1
Fast food	7	436	80.7	16.7	2.5	628	61.8	30.9	7.3	627	58.9	27.8	13.4
Sweetened soft drinks	7	436	64.9	23.4	11.7	628	50.8	26.8	22.5	628	41.1	32.0	26.9
Fruit or mixed fruit and vegetable juices	7	434	59.0	26.7	14.3	629	42.9	33.7	23.4	628	43.5	30.7	25.8
Energy drinks	7	437	98.4	1.4	0.2	629	80.1	12.4	7.5	628	83.9	8.4	7.6
Vegetables	7	437	66.6	19.9	13.5	628	45.9	32.3	21.8	627	46.3	26.3	27.4
Fruit	7	432	66.2	22.7	11.1	629	44.5	34.0	21.5	627	42.9	28.9	28.2
Sweets	7	433	70.2	21.5	8.3	629	49.4	36.1	14.5	628	43.8	31.1	25.2
pHDI	3	430	79.5	20.5	0	625	75.8	23.5	0.6	622	69.0	30.5	0.5
nHDI	3	432	96.8	3.2	0.0	625	92.0	7.7	0.3	623	92.3	7.7	0.0
Active/sedentary lifestyle													
Screen time	6	434	86.9	11.5	1.6	623	64.8	24.9	10.3	625	60.0	29.9	10.1
Physical activity level	3	432	86.3	13.4	0.2	622	74.8	24.6	0.6	626	65.3	32.1	2.6
Physical activity at school	3	435	84.4	14.5	1.1	622	74.6	24.0	1.4	627	64.6	29.8	5.6
Physical activity at leisure time	3	432	83.8	15.3	0.9	623	71.3	27.0	1.8	626	59.9	37.9	2.2
FAS components													
How many computers, laptops, or tablets does your family own?	4	433	87.1	11.5	1.4	626	83.7	15.2	1.1	623	75.6	20.5	3.9
Does your family own a car, van, or truck?	3	433	91.9	7.6	0.5	627	86.3	11.5	2.2	622	83.6	13.0	3.4
Does your family have a dishwasher?	2	435	97.5	2.5		625	95.8	4.2		622	95.5	4.5	
Do you have your own bedroom?	2	435	96.1	3.9		622	95.8	4.2		620	92.9	7.1	
How many bathrooms (room with a bath or shower) are in your home?	4	434	95.2	4.1	0.7	628	90.9	7.5	1.6	624	89.4	9.9	0.6
Does your home have an outdoor space attached (e.g., garden)?	2	432	95.1	4.9		625	95.2	4.8		622	95.2	4.8	
BMI-for-age	3	407	89.4	10.6	0.0	583	90.2	9.6	0.2	585	92.1	7.7	0.2

^1^ number of categories in the question; cat.—categories; nutrition knowledge level—based on 18 questions and assessed in three categories: Low (5 points), moderate (12 points), high (13–18 points) [22]; ^2^ in a group of 6–10-year-old children, nutrition knowledge was assessed in their parents; pHDI—a pro-healthy diet index composed of four questions (dairy products, fish, vegetables, fruit) and assessed in three categories: Low (0–33.32% points), moderate (33.33–66.65% points), high (66.6–100% points) [22]; nHDI—a non-healthy diet index composed of four questions (fast food, sweetened soft drinks, energy drinks, sweets) and assessed in three categories: Low (0–33.32% points), moderate (33.33–66.65% points), high (66.66–100% points) [22]; physical activity level—based on two questions: Physical activity at school and physical activity at leisure time and assessed in three categories: Low (0–1 points), moderate (2–4 points), high (5 points) [22]; FAS components—six questions of the Family Affluence Scale [26]; BMI-for-age—the age-sex-specific body mass index calculated using self-reported height and weight and assessed in three categories [27]; NA—not applied.

**Table 5:** Kappa statistics for test and retest of the SF-FFQ4PolishChildren questionnaire.

Variables	Cat. ^1^	Children Aged 6–10 Years	Adolescents Aged 11–15 Years	
		Parent (Test–Retest)	Adolescent (Test–Retest)	Adolescent (Test) & Parent (TestP)
Sample size		437	630	628
Nutrition knowledge level ^2^	3	0.62	0.36	NA
Dietary habits				
Breakfast	4	0.70	0.54	0.56
Meal at school	4	0.78	0.53	0.46
Dairy products	7	0.54	0.37	0.27
Fish	7	0.67	0.42	0.32
Fast food	7	0.68	0.43	0.33
Sweetened soft drinks	7	0.56	0.39	0.28
Fruit or mixed fruit and vegetable juices	7	0.46	0.30	0.30
Energy drinks	7	0.81	0.44	0.45
Vegetables	7	0.56	0.31	0.31
Fruit	7	0.53	0.31	0.27
Sweets	7	0.59	0.36	0.27
pHDI	3	0.55	0.44	0.31
nHDI	3	0.45	0.35	0.26
Active/sedentary lifestyle				
Screen time	6	0.72	0.46	0.35
Physical activity level	3	0.68	0.52	0.36
Physical activity at school	3	0.69	0.54	0.40
Physical activity at leisure time	3	0.69	0.51	0.31
FAS components				
How many computers, laptops, or tablets does your family own?	4	0.79	0.69	0.55
Does your family own a car, van, or truck?	3	0.84	0.76	0.70
Does your family have a dishwasher?	2	0.93	0.89	0.87
Do you have your own bedroom?	2	0.90	0.83	0.74
How many bathrooms (room with a bath or shower) are in your home?	4	0.88	0.84	0.79
Does your home have an outdoor space attached (e.g., garden)?	2	0.85	0.85	0.84
BMI-for-age	3	0.67	0.64	0.65

^1^ Cat.—number of categories in the question; nutrition knowledge level—based on 18 questions and assessed in three categories: Low (0–5 points), moderate (6–12 points), high (13–18 points) [22]; ^2^ in a group of 6–10-year-old children, nutrition knowledge was assessed in their parents; pHDI—a pro-healthy diet index composed of four questions (dairy products, fish, vegetables, fruit) and assessed in three categories: Low (0–33.32% points), moderate (33.33–66.65% points), high (66.66–100% points) [22]; nHDI—a non-healthy diet index composed of four questions (fast food, sweetened soft drinks, energy drinks, sweets) and assessed in three categories: Low (0–33.32% points), moderate (33.33–66.65% points), high (66.66–100% points) [22]; physical activity level—based on two questions: Physical activity at school and physical activity at leisure time, and assessed in three categories: Low (0–1 points), moderate (2–4 points), high (5 points) [22]; FAS components—six questions of the Family Affluence Scale [26]; BMI-for-age—the age-sex-specific body mass index calculated using self-reported height and weight and assessed in three categories [27]; NA—not applied.

**Table 6:** Correlation coefficients (*r*)^1^ between test and retest of the SF-FFQ4PolishChildren questionnaire.

Variables (All in Points)	Range of Points	Children Aged 6–10 Years (*n* = 437)	Adolescents Aged 11–15 Years (*n* = 630)	
		Parent (Test–Retest)	Adolescent (Test–Retest)	Adolescent (Test) & Parent (TestP)
Nutrition knowledge score ^2^	0–18	0.80	0.68	NA
pHDI	0–100	0.76	0.63	0.52
nHDI	0–100	0.83	0.68	0.53
Screen time score	0–5	0.78	0.58	0.45
Physical activity score	0–5	0.77	0.71	0.51
FAS	0–9	0.93	0.91	0.90

^1^*r*—the Spearman’s rank correlation coefficient (all <0.05); nutrition knowledge score—evaluated based on 18 questions [22]; ^2^ in a group of 6–10-year-old children, nutrition knowledge was assessed in their parents; pHDI—a pro-healthy diet index composed of four questions (dairy products, fish, vegetables, fruit) [22]; nHDI—a non-healthy diet index composed of four questions (fast food, sweetened soft drinks, energy drinks, sweets) [22]; screen time score—based on a single question with six response categories [22]; physical activity score—based on two questions: Physical activity at school and physical activity at leisure time [22]; FAS—the Family Affluence Scale composed of six questions [26]; NA—not applied.

## Supplementary Materials

The following are available online at https://www.mdpi.com/2072-6643/11/12/2929/s1, Figure S1: The academic centers (including cities and villages around) where data were collected, Table S1: Correlation coefficients (*r*) between test and retest of the SF-FFQ4PolishChildren questionnaire by sex and place of residence, Figure S2: Boys: Bland–Altman plots for the pro-healthy diet index (pHDI; left panel) and the non-healthy diet index (nHDI; right panel) between the first and the second administration of the questionnaire, Figure S3: Girls: Bland–Altman plots for the pro-healthy diet index (pHDI; left panel) and the non-healthy diet index (nHDI; right panel) between the first and the second administration of the questionnaire, Table S2: Means and 95% confidence interval (CI) in test and retest of the SF-FFQ4PolishChildren questionnaire in the total sample and by sex and place of residence.
