# Supplementary material for: Reproducibility of a Short-Form, Multicomponent Dietary Questionnaire to Assess Food Frequency Consumption, Nutrition Knowledge, and Lifestyle (SF-FFQ4PolishChildren) in Polish Children and Adolescents
Source: Nutrients. 2019 Dec 3;11(12):2929. doi: 10.3390/nu11122929 (PMC6950380; doi:10.3390/nu11122929)
Supplement: Supplementary file 1 [file nutrients-11-02929-s001.pdf]

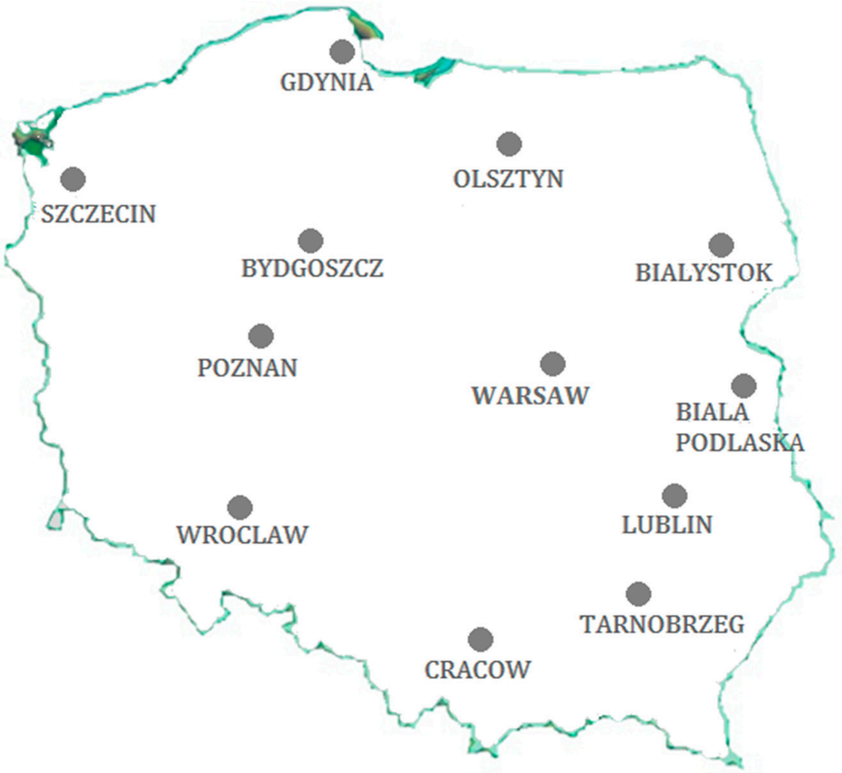

Figure S1. The academic centres (including cities and villages around) where data were collected [22].

Table S1. Correlation coefficients (*r*) [1] between test and retest of the SF-FFQ4PolishChildren questionnaire by sex and place of residence.

Variables (all in points)	Range of points	Children aged 6-10 years (*n* = 437)	Adolescents aged 11-15 years (*n* = 630)	
		Parent (test-retest)	Adolescent (test-retest)	Adolescent (test) & Parent (testP)
Boys				
Nutrition knowledge score [2]	0–18	0.81	0.66	NA
pHDI	0–100	0.75	0.60	0.52
nHDI	0-100	0.81	0.68	0.48
Screen time score	0–5	0.77	0.53	0.36
Physical activity score	0–5	0.74	0.69	0.51
FAS	0–9	0.91	0.90	0.87
Girls				
Nutrition knowledge score [2]	0–18	0.80	0.70	NA
pHDI	0–100	0.76	0.66	0.51
nHDI	0–100	0.83	0.67	0.57
Screen time score	0–5	0.78	0.62	0.54
Physical activity score	0–5	0.79	0.73	0.52
FAS	0–9	0.94	0.92	0.92
Rural residents				
Nutrition knowledge score [2]	0-18	0.85	0.64	NA
pHDI	0-100	0.78	0.61	0.57
nHDI	0-100	0.84	0.62	0.60
Screen time score	0-5	0.76	0.55	0.50
Physical activity score	0-5	0.76	0.69	0.51
FAS	0-9	0.89	0.83	0.88
Urban residents [3]				
Nutrition knowledge score [2]	0-18	0.75	0.72	NA
pHDI	0-100	0.72	0.65	0.47
nHDI	0-100	0.81	0.73	0.46
Screen time score	0-5	0.80	0.60	0.41
Physical activity score	0-5	0.79	0.72	0.52
FAS	0-9	0.96	0.95	0.88

[1] *r*—the Spearman's rank correlation coefficient (all <0.05); nutrition knowledge score—evaluated based on 18 questions [22]; [2] in a group of 6-10-year-old children nutrition knowledge was assessed in their parents; pHDI—a pro-Healthy Diet Index composed of 4 questions (dairy products, fish, vegetables, fruit) [22]; nHDI—a non-Healthy Diet Index composed of 4 questions (fast food, sweetened soft drinks, energy drinks, sweets) [22]; screen time score—based on a single questions with 6 response categories [22]; physical activity score—based on two questions: physical activity at school and physical activity at leisure time [22]; FAS—the Family Affluence Scale composed of 6 questions [26]; [3] urban residents—respondents who indicated one of the following categories of place of residence: 'town' or 'city (≥100 000 inhabitants)'; NA—not applied.

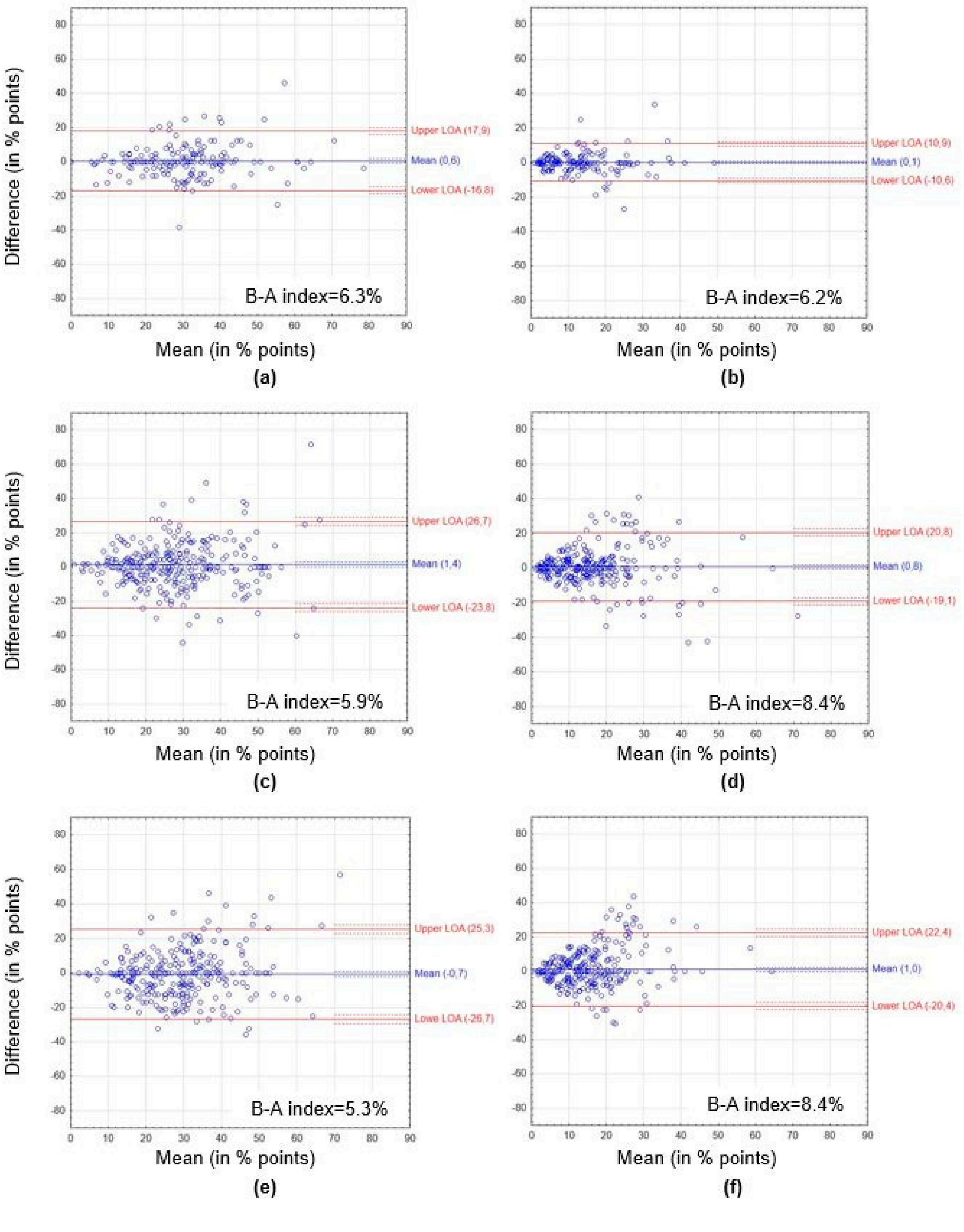

Figure S2. Boys: Bland-Altman plots for the pro-Healthy-Diet-Index (pHDI; left panel) and the non-Healthy-Diet-Index (nHDI; right panel) between the first and the second administration of the questionnaire: (**a**) pHDI in children aged 6-10 years (test and retest), (**b**) nHDI in children aged 6-10 years (test and retest), (**c**) pHDI in adolescents aged 11–15 years (test and retest), (**d**) nHDI in adolescents aged 11-15 years (test and retest), (**e**) pHDI in adolescents aged 11–15 years and their parents (test and testP), (**f**) nHDI in adolescents aged 11–15 years and their parents (test and testP). Mean—mean difference between the first and the second administration of the questionnaire (blue solid line) with 95% CI (dashed lines). LOA—95% limits of agreement between the first and the second administration of the questionnaire (red solid lines) with 95% CI (dashed lines). B-A index—the Bland-Altman index calculated as percentage of respondents beyond LOA. .

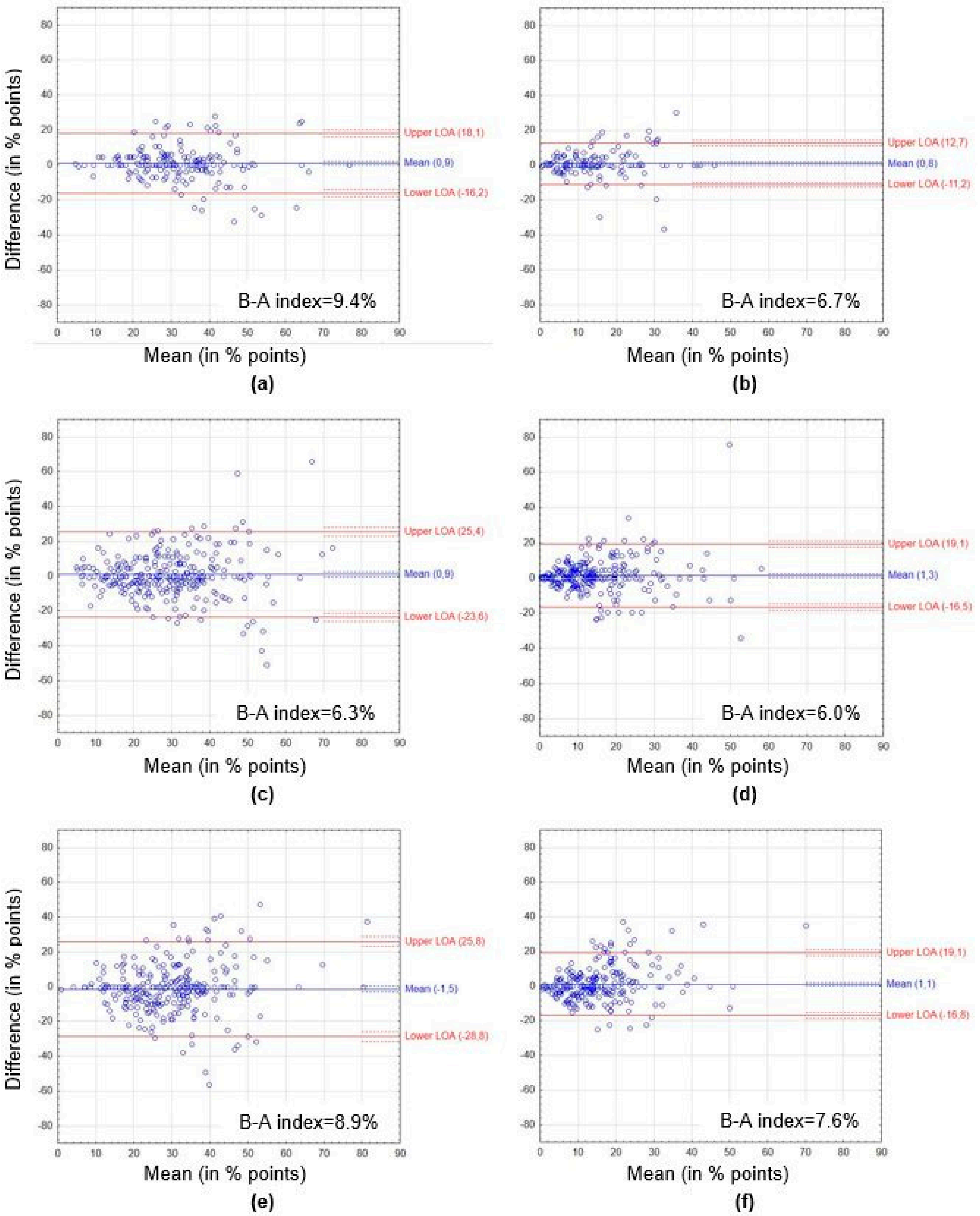

Figure S3. Girls: Bland-Altman plots for the pro-Healthy-Diet-Index (pHDI; left panel) and the non-Healthy-Diet-Index (nHDI; right panel) between the first and the second administration of the questionnaire: (**a**) pHDI in children aged 6-10 years (test and retest), (**b**) nHDI in children aged 6-10 years (test and retest), (**c**) pHDI in adolescents aged 11-15 years (test and retest), (**d**) nHDI in adolescents aged 11-15 years (test and retest), (**e**) pHDI in adolescents aged 11-15 years and their parents (test and testP), (**f**) nHDI in adolescents aged 11-15 years and their parents (test and testP). Mean—mean difference between the first and the second administration of the questionnaire (blue solid line) with 95% CI (dashed lines). LOA—95% limits of agreement between the first and the second

administration of the questionnaire (red solid lines) with 95% CI (dashed lines). B-A index—the Bland-Altman index calculated as percentage of respondents beyond LOA. .

Table S2. Means and 95% confidence interval (CI) in test and retest of the SF-FFQ4PolishChildren questionnaire in the total sample and by sex and place of residence.

Variables (all in points)	Range of points	Children aged 6-10 years (*n* = 437)			Adolescents aged 11-15 years (*n* = 630)				
		Test (parent)	Retest (parent)	*P* [1]	Test (adolescent)	Retest (adolescent)	*P* [1]	TestP (parent)	*P* [1]
Total sample									
Nutrition knowledge score[2]	0-18	10.4 (10.1; 10.6)	10.4 (10.1; 10.6)	ns	7.2 (6.9; 7.4)	7.5 (7.3; 7.7)	**	NA	
pHDI	0-100	31.5 (30.3; 32.8)	30.8 (29.6; 32.0)	ns	29.0 (27.9; 30.1)	27.8 (26.7; 28.9)	*	30.2 (29.1; 31.2)	**
nHDI	0-100	13.0 (12.2; 13.9)	12.6 (11.7; 13.4)	*	15.1 (14.2; 16.0)	14.0 (13.1; 14.9)	**	14.0 (13.2; 14.7)	ns
Screen time score	0-5	0.4 (0.4; 0.5)	0.5 (0.4; 0.5)	ns	0.9 (0.8; 1.0)	1.0 (0.9; 1.1)	*	0.7 (0.6; 0.8)	****
Physical activity score	0-5	3.2 (3.0; 3.3)	3.1 (3.0; 3.2)	ns	3.4 (3.3; 3.5)	3.3 (3.2; 3.4)	ns	3.0 (2.9; 3.1)	****
FAS	0-9	6.2 (6.0; 6.4)	6.2 (6.0; 6.4)	ns	6.8 (6.7; 6.9)	6.8 (6.6; 6.9)	ns	6.6 (6.5; 6.8)	***
Boys									
Nutrition knowledge score[2]	0-18	10.5 (10.1; 10.8)	10.6 (10.2; 10.9)	ns	6.9 (6.6; 7.2)	7.1 (6.7; 7.5)	ns	NA	
pHDI	0-100	30.5 (28.7; 32.4)	30.0 (28.3; 31.7)	ns	28.4 (26.8; 29.9)	26.9 (25.5; 28.4)	*	29.1 (27.8; 30.5)	ns
nHDI	0-100	12.6 (11.4; 13.8)	12.5 (11.3; 13.6)	ns	15.5 (14.2; 16.8)	14.7 (13.4; 16.0)	ns	14.5 (13.4; 15.5)	ns
Screen time score	0-5	0.4 (0.3; 0.5)	0.4 (0.4; 0.5)	ns	1.0 (0.9; 1.1)	1.1 (0.9; 1.2)	ns	0.7 (0.7; 0.8)	***
Physical activity score	0-5	3.4 (3.2; 3.6)	3.3 (3.1; 3.4)	*	3.4 (3.3; 3.6)	3.3 (3.1; 3.4)	*	3.0 (2.9; 3.2)	****
FAS	0-9	6.4 (6.2; 6.7)	6.4 (6.2; 6.7)	ns	6.8 (6.7; 7.0)	6.8 (6.6; 7.0)	ns	6.6 (6.4; 6.8)	***
Girls									
Nutrition knowledge score[2]	0-18	10.3 (9.9; 10.6)	10.2 (9.8; 10.6)	ns	7.5 (7.1; 7.8)	7.9 (7.6; 8.2)	*	NA	
pHDI	0-100	32.5 (30.9; 34.1)	31.6 (29.9; 33.2)	ns	29.7 (28.1; 31.3)	28.8 (27.2; 30.3)	ns	31.3 (29.8; 32.8)	*
nHDI	0-100	13.4 (12.1; 14.7)	12.7 (11.5; 13.9)	**	14.6 (13.3; 15.9)	13.3 (12.1; 14.5)	**	13.4 (12.4; 14.5)	ns
Screen time score	0-5	0.5 (0.4; 0.5)	0.5 (0.4; 0.6)	ns	0.8 (0.7; 0.9)	0.9 (0.8; 1.0)	*	0.6 (0.5; 0.7)	***

Variables (all in points)	Range of points	Children aged 6-10 years (*n* = 437)			Adolescents aged 11-15 years (*n* = 630)				
		Test (parent)	Retest (parent)	*P* [1]	Test (adolescent)	Retest (adolescent)	*P* [1]	TestP (parent)	*P* [1]
Physical activity score	0-5	2.9 (2.7; 3.1)	2.9 (2.7; 3.1)	ns	3.3 (3.2; 3.5)	3.3 (3.2; 3.5)	ns	3.0 (2.8; 3.1)	****
FAS	0-9	6.0 (5.7; 6.2)	6.0 (5.8; 6.2)	ns	6.7 (6.5; 6.9)	6.7 (6.5; 6.9)	ns	6.6 (6.4; 6.8)	ns
Rural residents									
Nutrition knowledge score[2]	0-18	10.2 (9.8; 10.6)	10.0 (9.6; 10.4)	ns	7.2 (6.9; 7.5)	7.1 (6.7; 7.5)	ns	NA	
pHDI	0-100	31.7 (29.9; 33.5)	31.3 (29.5; 33.0)	ns	29.5 (28.0; 31.1)	27.3 (25.8; 28.8)	***	30.0 (28.4; 31.7)	ns
nHDI	0-100	14.3 (12.9; 15.7)	13.7 (12.4; 15.0)	ns	15.2 (14.0; 16.5)	15.0 (13.6; 16.4)	ns	14.6 (13.4; 15.7)	ns
Screen time score	0-5	0.4 (0.4; 0.5)	0.5 (0.4; 0.6)	ns	0.9 (0.8; 1.0)	1.0 (0.9; 1.2)	ns	0.7 (0.6; 0.8)	***
Physical activity score	0-5	3.1 (2.9; 3.3)	3.0 (2.8; 3.2)	*	3.4 (3.3; 3.6)	3.3 (3.1; 3.4)	*	3.0 (2.8; 3.1)	****
FAS	0-9	6.6 (6.4; 6.9)	6.6 (6.3; 6.8)	ns	7.3 (7.1; 7.5)	7.2 (7.1; 7.4)	ns	7.2 (7.0; 7.4)	ns
Urban residents [3]									
Nutrition knowledge score[2]	0-18	10.5 (10.1; 10.9)	10.8 (10.4; 11.1)	*	7.1 (6.8; 7.5)	7.8 (7.5; 8.1)	****	NA	
pHDI	0-100	31.3 (29.7; 33.0)	30.3 (28.7; 31.9)	ns	28.5 (27.0; 30.1)	28.3 (26.8; 29.8)	ns	30.3 (29.0; 31.6)	**
nHDI	0-100	11.6 (10.6; 12.6)	11.3 (10.2; 12.3)	ns	14.9 (13.6; 16.2)	13.1 (12.0; 14.3)	**	13.4 (12.5; 14.4)	ns
Screen time score	0-5	0.4 (0.3; 0.5)	0.4 (0.3; 0.5)	ns	0.9 (0.8; 1.0)	0.9 (0.8; 1.1)	ns	0.6 (0.6; 0.7)	***
Physical activity score	0-5	3.2 (3.0; 3.4)	3.2 (3.0; 3.4)	ns	3.4 (3.2; 3.5)	3.3 (3.2; 3.5)	ns	3.0 (2.9; 3.2)	****
FAS	0-9	5.7 (5.5; 5.9)	5.8 (5.5; 6.0)	ns	6.4 (6.2; 6.5)	6.4 (6.2; 6.6)	ns	6.1 (5.9; 6.3)	**

[1] *P*—significance level of Wilcoxon's test: *<0.05, **<0.01, ***<0.001, ****<0.0001; nutrition knowledge score—evaluated based on 18 questions [22]; [2] in a group of 6-10-year-old children nutrition knowledge was assessed in their parents; pHDI—a pro-Healthy Diet Index composed of 4 questions (dairy products, fish, vegetables, fruit) [22]; nHDI—a non-Healthy Diet Index composed of 4 questions (fast food, sweetened soft drinks, energy drinks, sweets) [22]; screen time score—based on a single questions with 6 response categories [22]; physical activity score—based on two questions: physical activity at school and physical activity at leisure time [22]; FAS—the Family Affluence Scale composed of 6 questions [26]; [3] urban residents—respondents who indicated one of the following categories of place of residence: 'town' or 'city (≥100 000 inhabitants)'; NA—not applied.
